# Enhanced Control of *Leucoptera coffeella* on Coffee Leaves Using Cyantraniliprole-Hybrid Polymeric Membranes

**DOI:** 10.1021/acsomega.5c11821

**Published:** 2026-01-23

**Authors:** Caroline Nunes dos Reis, Lorena Alves de Melo Bessa, Maria Gabrielle Silva, Thaissa Moreira Santos, Keyller Bastos Borges, Eduardo Alves, Júlio César José da Silva, Gustavo Franco de Castro, Carlos Gustavo da Cruz, Flávio Lemes Fernandes, Jairo Tronto

**Affiliations:** † Instituto de Ciências Exatas e Tecnológicas, Universidade Federal de Viçosa, Campus Rio Paranaíba, 38810-000 Rio Paranaíba, Minas Gerais, Brazil; ‡ Departamento de Ciências Naturais, 74383 Universidade Federal de São João del-Rei (UFSJ), Campus Dom Bosco, Praça Dom Helvécio 74, Fábricas, 36301-160 São João del-Rei, Minas Gerais, Brazil; § Universidade Federal de Lavras, Campus Lavras, 37200-000 Lavras, Minas Gerais, Brazil; ∥ Departamento de Química Instituto de Ciências Exatas (ICE), 28113 Universidade Federal de Juiz de Fora (UFJF), Campus Universitário, s/n, Bairro Martelos, 36036-900 Juiz de Fora, Minas Gerais, Brazil; ⊥ Universidade Federal de Viçosa, Campus Viçosa, 36570-900 Viçosa, Minas Gerais, Brazil

## Abstract

*Leucoptera coffeella* is a significant lepidopteran pest of coffee (*Coffea* spp.) crops, capable of causing yield losses of up to 80%. While chemical control with synthetic insecticides remains the predominant strategy, challenges such as pest resistance and environmental contamination highlight the urgent need for more sustainable alternatives. Hybrid polymeric membranes, formulated from Laponite RD clay, sodium alginate, and the insecticide cyantraniliprole, were developed in this study to serve as protective coatings for coffee leaves. Their successful synthesis and the effective integration and interaction of cyantraniliprole within the hybrid matrix were corroborated by proper characterization, including powder X-ray diffraction, Fourier-transform infrared spectroscopy with attenuated total reflectance, thermogravimetric analysis coupled with differential scanning calorimetry, and scanning electron microscopy with energy-dispersive spectroscopy. Bioassay experiments in a greenhouse using seedlings of Catuaí vermelho, a coffee variety of the *Coffea arabica* species, were conducted in a randomized block design with eight treatments and four replicates. Larval mortality and egg deposition were assessed and statistically analyzed using the Scott-Knott test (*p* < 0.05). The hybrid membranes significantly increased larval mortality and reduced oviposition compared to both control and commercial insecticide treatments. These findings underscore the potential of these membranes as an eco-friendly alternative for integrated pest management in coffee cultivation, offering advantages such as improved adhesion, sustained release, and reduced pesticide usage.

## Graphical abstract

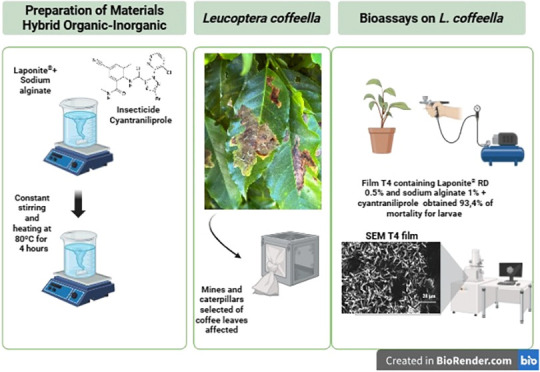

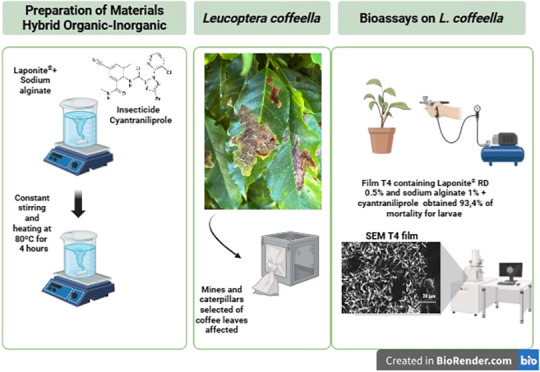

## 1 Introduction

Coffee (*Coffea* spp.) is one of the world’s most widely consumed agricultural products. Its trade is highly intricate and profitable despite being classified as a nonessential food commodity. Brazil is the world’s leading coffee producer, accounting for approximately 38% of total production. For the 2025 harvest, estimates project production of 55.2 million processed bags, with 35.2 million bags of arabica coffee and 20.1 million bags of conilon coffee.^1^ Coffee cultivation plays a significant role in Brazil’s social and economic landscape, especially in rural development.

Numerous studies have been conducted to enhance coffee production, with a primary focus on addressing phytosanitary issues caused by pests, such as the coffee leaf miner *Leucoptera coffeella* (Lepidoptera: Lyonetiidae).^2^ This insect is a significant threat to coffee plants, with its larvae feeding on the inner tissue of the leaves, causing damage that reduces photosynthetic capacity and results in defoliation of the plantation.^2^ Depending on the severity of infestation, losses can reach up to 70% of production.^3^ It is important to use effective control methods to reduce the damage caused by this pest. Chemical control remains a prevalent method among coffee growers. The main insecticides employed are organophosphates, pyrethroids, and carbamates, all of which exhibit a broad spectrum of activity.^1,2^ Nonetheless, the nonselective nature of these products raises significant environmental concerns. A more recent chemical group, the anthranilic diamides, has emerged and gained popularity due to its favorable environmental profile and minimal impact on nontarget insect species.^4^ This class of insecticides acts on insect ryanodine receptors, disrupting calcium homeostasis in muscle cells, which causes sustained muscle contraction and subsequent paralysis.

While chemical control is currently the most efficacious method, its injudicious application can severely hinder effective pest management if proper rotation of active ingredients or chemical groups is neglected.^5^ This practice introduces several challenges, notably the diminished efficacy and selectivity of insecticides, imprecise delivery of the active ingredient to the target pest, and the subsequent evolution of pest resistance. Consequently, the development of novel control methodologies is vital. These methods must both incorporate chemical approaches and implement strategies to re-establish environmental equilibrium, all while ensuring economic viability for agricultural producers. Furthermore, pesticide application technologies can profoundly impact pest control decisions, with spray volume being a central focus of application research. Nevertheless, reduced spray volumes present a critical hurdle in achieving effective pest control: ensuring uniform droplet distribution. Since *L*. *coffeella* larvae are protected within leaf mines, achieving direct physical contact with applied agricultural pesticides is inherently difficult.^6^ Consequently, to maximize product deposition onto the larvae, conventional applications often involve liquid volumes that surpass the maximum retention capacity of the foliage. Therefore, a pressing demand exists for the development of advanced materials possessing enhanced properties. These innovations must serve the dual purpose of boosting agricultural productivity and contributing to environmental sustainability.

In this context, organic–inorganic hybrid materials have been the subject of numerous studies due to their unique properties compared to their isolated precursors.^7^ These materials are formed by the proper combination of organic and inorganic components, whose synergistic interaction results in materials with unique properties not found in conventional materials.^8^ The preparation of organic–inorganic hybrid materials in agriculture has been widely studied for their prophylactic potential in pest and disease management.^9^ Nanosilica has been also for different application, including to control chicken malaria and the nuclear polyhedrosis virus (BmNPV), a scourge of the silk industry.^10^ Insects possess cuticular lipids that protect them from water contact, preventing death by desiccation. Nano silicas are absorbed by cuticular lipids through physisorption, causing insect death through physical contact. Hybrid materials, composed of carboxymethyl starch and sodium montmorillonite, have also been investigated for their application as seed tapes in agriculture.^11^ These multilayered tapes, which encapsulate seeds between at least two distinct layers, exploit the inherent properties of their constituent materials to promote seed germination. This is achieved by effectively retaining moisture and establishing ideal environmental conditions conducive to robust seedling development.

Although some studies have been published on using organic–inorganic hybrid materials for seed coating in agriculture, the application of such materials for leaf coating has not yet been fully explored. These materials may offer several advantages over pure materials, such as a greater capacity for swelling (absorbing large amounts of water), creating a more effective gas barrier, and providing physical protection when applied as protective membranes. This physical protection can also influence the rate of insecticide release, prolong the active ingredient’s action, and reduce the number of applications. In addition to improving the above-mentioned properties, these materials may offer lower production costs. Therefore, as novelty this work aimed to prepare and characterize organic–inorganic hybrid materials derived from the interaction between Laponite RD (Lap) and sodium alginate (Alg) by incorporating cyantraniliprole insecticide, which were applied as membranes on coffee leaves. The action of the hybrid membranes in controlling *L*. *coffeella* has been also studied by bioassays conducted in a greenhouse.

## 2 Experimental Section

### 2.1 Reagents

The following reagents were used to produce polymeric membranes: Laponite – Lap (100%, Buntech, São Paulo, SP, Brazil), Sodium alginate – Alg (90.8–106%, Êxodo Científica Sumaré, SP, Brazil), and cyantraniliprole (95%, Sigma-Aldrich, Germany). The ultrapure water has been obtained through the MiliQ system (Millipore, France).

### 2.2 Preparation of Suspension Gels

#### 2.2.1 Preparation of Laponite Gels

All Laponite (Lap) was suspended in ultrapure water at concentrations of 0.5, 1.0, 2.0, and 3% (w/v). The suspensions were prepared in a condensation system connected to flasks and kept under constant stirring and heating at 80 °C until complete homogenization. This process was necessary for the exfoliation of the clay. Then, the suspension was cooled until gel formation.^12^ The resulting gel was reserved to be added to the sodium alginate solution described later.

#### 2.2.2 Preparation of Suspensions and Gels Containing Cyantraniliprole

The preparation of Lap suspensions containing cyantraniliprole 100 OD (FMC company, 95%, Sigma-Aldrich) was carried out by incorporating the clay in a solution containing the insecticide. This solution was previously prepared with the concentration recommended by the insecticide manufacturer to control *L*. *coffeella* in coffee production (0.0978 mg L^–1^). Gels were prepared at different concentrations: 0.5, 1.0, 2.0, 3.0% (w/v), which were maintained in a condensation system under constant stirring and heating at 80 °C for 4 h to ensure complete exfoliation of the clay. Then, the resulting gel was reserved to be later added to the sodium alginate solution described later.

#### 2.2.3 Preparation of Polymeric Membranes

Different organic–inorganic hybrid membranes have been obtained by variation of Lap/Alg ratios (Table S1). To prepare Lap/Alg membranes, the previously prepared Lap gels were added to the Alg solution, solubilized in ultrapure water at a concentration of 0.5, 1.0, and 2.0% (w/v), under constant stirring until complete solubilization. After preparation, the gels were applied to a Petri dish to obtain polymeric membranes. For the preparation of Lap/Alg membranes containing cyantraniliprole, gels were individually prepared in cyantraniliprole 100 OD aqueous solution at the concentration recommended by the insecticide manufacturer (FMC Company, São Paulo) (0.0978 mg L^–1^) for the control of *L*. *coffeella*, as described previously. These mixtures were kept under constant stirring for 1 h and then stored in hermetically sealed tubes for field application.

### 2.3 Structural Characterization of the Membranes

For the powder X-ray diffraction (PXRD) analysis, a Shimadzu XRD-6000 X-ray diffractometer was used. This equipment includes a graphite crystal monochromator, which selects the Cu–Kα1 radiation with a wavelength of 1.5406 Å. The source’s electrical current was set to 30 mA, and the potential was set to 30 kV. The resulting diffractograms from the analysis were obtained over a scanning range (2θ) from 4 to 70°, with a scanning interval of 1.0° per min. Fourier Transform Infrared Spectroscopy with Attenuated Total Reflectance (FTIR-ATR) analysis was performed using a Jasco FTIR 4100 spectrophotometer equipped with an ATR accessory. The spectra were recorded over a wavelength range of 4000 to 400 cm^–1^. Thermogravimetric analyses were carried out using an SDT Q600 V20.9 Build 20 instrument, a standard DSC-TGA model capable of performing simultaneous TG-DSC analyses. The heating program used ranged from room temperature to 1000 °C, with a heating rate of 10 °C min^–1^ in an atmosphere of synthetic air, with a flow rate of 100 mL min^–1^. The morphology of the materials was analyzed using a scanning electron microscope, model CLARA EVO 40XVP (Carl Zeiss SMT), operating at a voltage of 10 kV. The samples were mounted on carbon double-sided adhesive tape, previously attached to sample holders (stubs). The samples were then coated with carbon using a carbon evaporator (BAL-TEC, 1994). Energy-dispersive spectroscopy analyses were performed on the same equipment to characterize the samples chemically.

### 2.4 Coating Test

The test consisted of applying membranes with different Lap/Alg ratios onto water-sensitive paper (26 × 76 mm, Syngenta, Switzerland) to verify the quality of the droplet dispersion and uniformity, as the paper is sensitive to water, and the areas hit by the spray solution turn blue, aiding in the visualization of the coating. The application used a sprayer with a lack-110°/02 type nozzle (gallons min^–1^) and carbonic gas as purge. During the application, the carbonic gas pressure was 60 pounds inch^–2^, timing the dispersion process. The area covered by the polymeric membrane on the water-sensitive paper was obtained using the ImageJ program, and the test was conducted in triplicate.

### 2.5 *L*. *coffeella* Rearing

Leaves were collected from red Catuaí coffee plants and placed in paper bags (30 × 40 × 60 cm) and transferred to the laboratory within the next 96 h. Those leaves with undamaged mines (without openings or signs of parasitism/predation) were selected for insect colony production. Leaves were maintained in wooden cages (55 × 60 × 90 cm) at 25 ± 1 °C, 70% relative humidity, and a 12:12 (light) photoperiod until adult emergence. Adults from each population were then transferred to wooden cages (100 × 100 × 200 cm) containing coffee plant seedlings (Catuaí IAC-144) potted inside a black pouch (22 cm in height × 8 cm in diameter) containing a 1:1 mixture of soil and composted cow manure and placed in a greenhouse at temperature 26 ± 1 °C, 70 ± 5% relative humidity without insecticide applications. A code name was assigned to each population for future identification.

The rearing of *L*. *coffeella* was conducted in 50 × 50 × 50 cm cages covered with antiaphid mesh and lined with moistened flannels to prevent the desiccation of the pupae and facilitate adult emergence. *L*. *coffeella* population was obtained by collecting leaves containing larvae and pupae from the experimental field of the Universidade Federal de Viçosa, Rio Paranaíba *Campus* (19°13′7.62″ S and 46°13′ 30.55″ W). The selection and collection area were determined by the low use of insecticides (three applications per year were conducted using spinosad (480 CS) at 125 mL ha^–1^ (on the leaf), deltamethrin (25 EC) at 400 mL ha^–1^ (on the leaf), and thiamethoxam (250 WG) at 100 g ha^–1^ (soil)) in these experimental fields, which favors obtaining fewer resistant populations. The collected leaves containing pupae and larvae were placed in the cages until adult emergence. After adult emergence, they were transferred to a cage covered with antiaphid mesh (300 × 200 × 120 cm), containing *C*. *arabica* seedlings for oviposition and population multiplication.

### 2.6 Toxicity Bioassays on *L*. *coffeella*

The experiment was conducted in a greenhouse located on the *Campus* of the Universidade Federal de Viçosa, in the city of Rio Paranaíba (19° 13′ 7.62″ S and 46° 13′ 30.55″ W) under temperature conditions of 23–30 °C and relative humidity of 50–65%. Coffee seedlings of the Paraíso II cultivar, with three pairs of leaves and without the presence of *L*. *coffeella* mines were used. The application was carried out using an airbrush (pen-type model) applying an average of 6 mL of solution to each pair of coffee leaves, ensuring complete coverage without runoff. After the application, a 30 min interval was timed to allow the product to dry completely, ensuring no interference. This time was established based on the longest drying process of seedlings. The control treatment was done using the same amount of solution but with distilled water. The seedlings were placed in a 300 × 200 × 120 cm cage with an antiaphid mesh. They were then infested with 80 insects for 2 days. Initially, egg evaluation was carried out after 2 days of infestation, reaching the proposed number of 30 eggs after 5 days. After 7 days, the mines on the leaves were evaluated, and after five more days, the mines were re-evaluated. After 10 days, the larval evaluation was performed. The coffee leaves were maintained in a laboratory set at 25 ± 1 °C, 65 ± 5% relative humidity, and 12:12 h light: dark until the mortality evaluation. The results showed that their mortality values (%) were corrected by the mortality (%) of the control (water), according to Abbott’s formula [Disp-formula eq1] ^13^ where, MC = corrected mortality (%); Mo = observed mortality of larvae; Mt = mortality of larvae in the control treatment.

1 MC=((Mo−Mt))/((100−Mt))×100

Mortality data were subjected to analysis of variance (ANOVA), and the means were compared using the Scott-Knott test at a 5% probability level using the Speed Stat 3.2 program.

## 3 Results and Discussion

### 3.1 Coating Test

Based on coating test, which is made to calculate the coated area and observe the homogeneity of the application, the polymeric membranes with different Lap/Alg rations were applied to water-sensitive paper to determine which would provide the best dispersion homogeneity. The tests were performed in triplicate, and the images are shown in Figure 1. Based on the area results, three polymeric membrane compositions were selected according to their coverage: low (<50%), medium (50–80%), and high (>80%). As showed in Figure 1, Lap 0.5% + Alg 1.0% presented 57.5% coverage, Lap 1.0% + Alg 1.0% presented 86.1% coverage, and Lap 2.0% + Alg 1.0% presented 35.2% coverage, which were coded as T1, T2, and T3, respectively. These Lap/Alg rations were selected for the next studies. All composition of polymeric membranes studied were coded as shown in Table 1.

**Figure 1:**
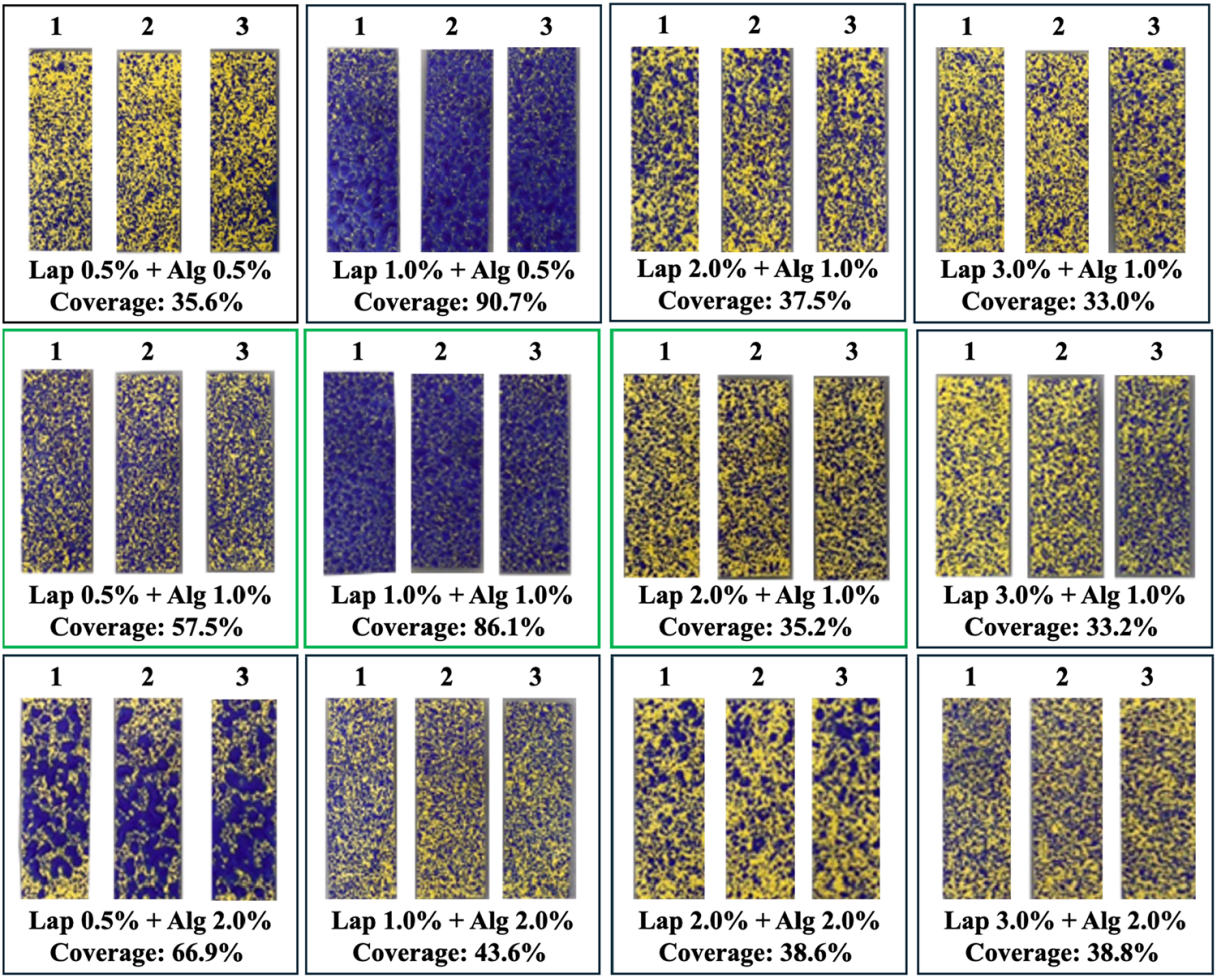
Water-sensitive papers coated with polymeric membranes with different Laponite RD/alginate ratios. The rations framed in green were selected for the studies.

**Table 1:** Description of Coffee Seedling Treatments Using Different Alginate and Laponite RD-Based Membranes

code	treatment description
T1	Lap 0.5% + Alg 1.0% medium (50–80%)
T2	Lap 1.0% + Alg 1.0% high (>80%)
T3	Lap 2.0% + Alg 1.0% low (<50%)
T4	Lap 0.5% + Alg 1.0% + cyantraniliprole
T5	Lap 1.0% + Alg 1.0% + cyantraniliprole
T6	Lap 2.0% + Alg 1.0% + cyantraniliprole
T7	cyantraniliprole
T8	control

Subsequently, an experimental design was employed, utilizing a randomized block design with eight treatments and four replications to be applied to coffee seedling leaves. Each replication corresponded to one pair of leaves, as illustrated in Figure 2. The treatments were also performed using the analytical standard of cyantraniliprole, as described in Table 1. The polymeric membranes were separated for further characterization and the leaves taken for bioassays experiments.

**Figure 2:**
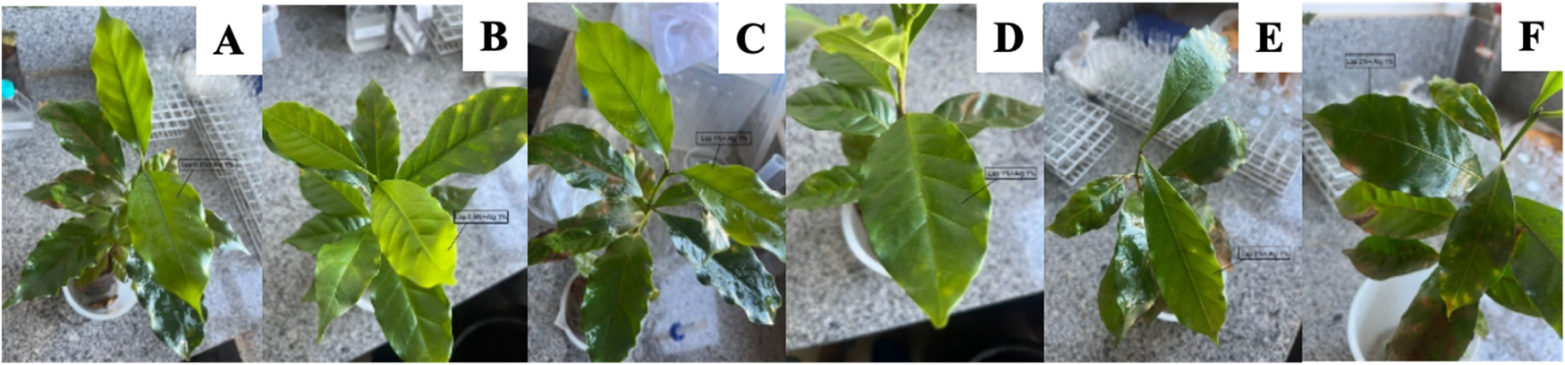
Application of polymeric membranes on coffee seedlings: (A) seedling coated with Lap 0.5% + Alg 1.0% treatment, photo taken immediately after application; (B) seedling coated with Lap 0.5% + Alg 1.0% treatment, photo taken 30 min after application; (C) seedling coated with Lap 1.0% + Alg 1.0% treatment, photo taken immediately after application; (D) seedling coated with Lap 1.0% + Alg 1.0% treatment, photo taken 30 min after application; (E) seedling coated with Lap 2.0% + Alg 1.0% treatment, photo taken immediately after application; (F) seedling coated with Lap 2.0% + Alg 1.0% treatment, photo taken 30 min after application.

### 3.2 Powder X-ray Diffraction (PXRD)

Figure 3 shows, respectively, the diffractograms of cyantraniliprole (black line), Laponite RD (orange line), sodium alginate biopolymer (blue line), and the polymeric membranes prepared only with the precursors and the precursors with insecticide cyantraniliprole at 0.0978 mg L^–1^ (T1 and T4: green line; T2 and T5: magenta line; T3 and T6: red line).

**Figure 3:**
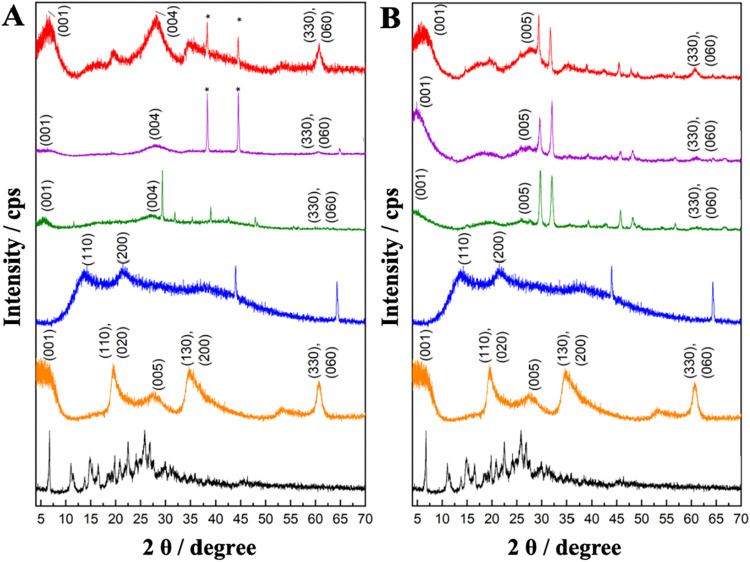
PXRD patterns of (A) cyantraniliprole (black line), clay Lap (orange line), sodium alginate (blue line), T1 (green line), T2 (magenta line), T3 (red line); (B) cyantraniliprole (black line), clay Lap (orange line), sodium alginate (blue line), T4 (green line), T5 (magenta line), T6 (red line). See Table 1 for T1–T3 for polymeric membranes and T4–T6 for polymeric membranes + cyantraniliprole.

The diffractogram of cyantraniliprole in Figure 3 (black line) shows crystalline peaks between 10 and 30° in the 2θ region, characteristic of the structure of the active ingredient.^14^ The diffractogram of the clay Lap (orange line) presents diffraction peaks in the regions 2θ = 6.70, 19.9, 28.3, 35.1, 53.7 and 61.0°, corresponding to the atomic planes (*hkl*) (001), (110, 020), (004), (130, 200), (150, 240) and (060, 330), respectively. The peak near 6.70° in the 2θ region is associated with the presence of Na^+^ ions (2θ = 6.75°; d001 = 1.31 Å). The peak at 2θ = 61.0° corresponding to the planes (*hkl*) (060,330) indicated the presence of a trioctahedral structure, typical of clay. In Figure 3 (blue line), the Alg biopolymer presents diffraction peaks of 13.5° and 22.0° in the 2θ region due to the hydrogen bonding interactions between the chains corresponding to the diffraction planes (110) and (200), respectively.

In Figure 3A (green, magenta, and red lines) corresponding to the polymeric membranes prepared with Lap/Alg biopolymer, the repetition of the diffraction peaks (001) in the region 2θ = 6.70° and (004) in the region 2θ = 28.3° is observed, indicating the presence of cationic clay. In Figure 3B (green, magenta, and red lines) corresponding to the polymeric membranes containing precursors and the insecticide cyantraniliprole at 0.0978 mg L^–1^, two crystalline peaks are formed in the 2θ region between 25° and 35°, which may be related to the presence of cyantraniliprole.

In the PXRD patterns of the hybrid membranes containing cyantraniliprole, additional crystalline reflections in the 2θ region of 25–35° (Figure 3B) suggest physical interactions between the active ingredient and the hybrid matrix. These diffractions were not observed in the individual components, indicating that the presence of cyantraniliprole may induce structural organization within the membrane, since in the molecule of the insecticide chlorantraniliprole, from the same family, peaks were observed in the 2θ region between 30.14 and 30.42°.^14^ It was possible to analyze the crystallographic phases of the prepared organic–inorganic hybrid materials and the exfoliation process of Lap clay.^15^ In the clay, the basal spacing calculated by the Bragg equation (nλ = 2d sin θ) was 1.38 nm, which confirms its lamellar structure.^16^ The low organization of the stacking axis of the clay layers and the strong hydrophilic affinity of the material resulted in a broad peak corresponding to the basal spacing.^17^ This effect occurs due to the formation of a pseudo layer of water molecules (approximately 2.5 Å thick) that surrounds the cations in the interlayer space, resulting in a detectable increase in basal spacing.^18^ The Alg biopolymer, in turn, exhibits broad diffraction peaks that demonstrate the partial crystallinity of the biopolymer structure.

The diffractograms of the prepared polymeric membranes, in Figure 3A,B (green, magenta, and red lines), showed a reduction in the intensity of the peak’s characteristic of the precursor materials. This can be attributed to the dilution effect of the polymeric matrix and the interaction between the constituent precursors of the polymeric membranes.^19^ The presence of the peak corresponding to the diffraction of the atomic plane (001) of Lap clay at the beginning of the spectra of the prepared membranes indicates partial exfoliation of the clay due to the low structural organization.^20^ Furthermore, the absence of other peaks indicates effective dispersion of the exfoliated nanomaterial in the polymer, and the shifts of the clay diffraction peaks suggest intercalation of Alg in the Lap layers.^21^

The diffraction profile of cyantraniliprole was like that of chlorantraniliprole, a molecule of the same insecticide family, with highly crystalline peaks. Although no study presents the interaction between the polymeric membrane and the insecticides, the peaks at 29.4 and 31.7° in the spectra of the prepared membranes (Figure 3B – green, magenta and red lines) suggest insecticide-precursor interaction.^14^ The peaks in the 2θ region between 35 and 50° in the diffractograms in Figure 3A (green, magenta and red lines) can be related to presence of metallic aluminum from the equipment’s sample holder.

### 3.3 Fourier Transform Infrared Spectroscopy with Attenuated Total Reflectance (FTIR-ATR)

Figure 4 shows the spectra of cyantraniliprole (Figure 4A), Lap clay (Figure 3B), Alg biopolymer (Figure 4C), and the polymeric membranes prepared with the precursors, i.e., T1 (Figure 4D), T2 (Figure 4E), T3 (Figure 4F), and with precursors and the insecticide cyantraniliprole at 0.0978 mg/L, i.e., T4 (Figure 4G), T5 (Figure 4H), and T6 (Figure 4I).

**Figure 4:**
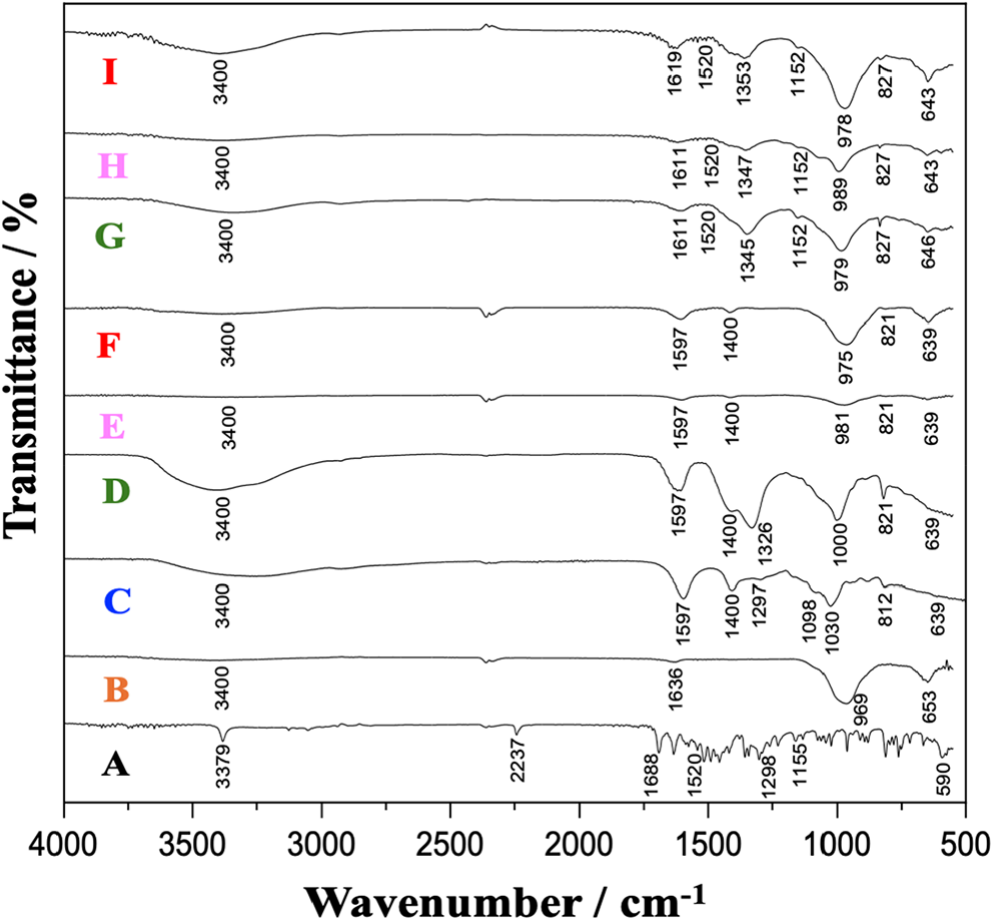
FTIR-ATR spectra for: (A) cyantraniliprole; (B) laponite RD; (C) sodium alginate; (D) T1 – Laponite RD 0.5% + sodium alginate 1.0%; (E) T2 – Laponite RD 1.0% and sodium alginate 1.0%; (F) T3 – Laponite RD 2% and sodium alginate 1.0%; (G) T4 – Laponite RD 0.5% and sodium alginate 1.0% + cyantraniliprole; (H) T5 – Laponite RD 1.0% and sodium alginate 1.0% + cyantraniliprole; (I) T6 – Laponite RD 2.0% and sodium alginate 1.0% + cyantraniliprole.

FTIR-ATR spectra of cyantraniliprole (Figure 4A) shows a stretching vibration of N–H single bonds at 3379 cm^–1^. At 2237 cm^–1^, the presence of nitriles in the insecticide structure is confirmed. The medium intensity band at 1688 cm^–1^ suggests the presence of C=O groups in amides and vibrations of N–H bonds. The band at 1520 cm^–1^ suggests the presence of N–H angular deformation vibrations, and at 1298 cm^–1^, of C–N bonds in an aromatic ring.^14^ A band at 590 cm^–1^ corresponds to the presence of bromide in the insecticide structure. In the FTIR-ATR spectrum of Lap (Figure 4B), a broad band in the region of 3400 cm^–1^ corresponds to the stretching vibrations of the O–H groups, which are associated with the adsorbed water molecules and the Si–OH groups, present in lamellae.^22^ A band of strong intensity at 969 cm^–1^ is associated with the stretching vibrations of the Si–O bonds in the clay structure. The band at 652 cm^–1^, of moderate intensity, indicates stretching vibrations characteristic of the Si–O bonds and bending vibrations of the Mg–OH-Mg bonds in structure.^23^ The spectrum of the biopolymer Alg (Figure 4C) shows a band of medium intensity in the region of 3400 cm^–1^, corresponding to the O–H bonds in the polymer structure.

The bands at 1597 and 1400 cm^–1^ indicate the carboxylate ions’ antisymmetric and symmetric vibrational modes. At 1030 and 812 cm^–1^, bands characteristic of the guluronic acid monomers in the Alg structure are observed.^22^ Figure 4D–4F shows the spectra of the polymeric membranes prepared with the organic–inorganic precursors. In these spectra, bands characteristic of the presence of Lap clay and Alg biopolymer are repeated, confirming the presence and interaction of the precursors. The FTIR spectra of the polymeric membranes loaded with cyantraniliprole (Figure 4G–4I) revealed characteristic bands of Lap and Alg, alongside two new distinct vibrational features. These included a band at 1520 cm^–1^, assigned to the N–H angular deformation, and a bending band at 1152 cm^–1^, indicative of primary amide functional groups.^14^ Both may be related to the presence and interaction of the precursors with the insecticide cyantraniliprole. Notably, the characteristic bands of Lap remained unchanged in the hybrid membranes, indicating that the clay structure was preserved. This suggests that the interaction between Lap and cyantraniliprole occurs primarily through surface adsorption rather than intercalation into the clay layers. This interpretation is coherent with the PXRD results, where no shift in the basal reflection (001) of Lap has been detected.

Therefore, FTIR-ATR allowed the analysis of the specific vibrational modes of the constituent functional groups of the prepared membranes. Some bands present slight shifts, which can be justified by the interaction between the precursors.^19^ The characteristic bands of Lap clay and the Alg biopolymer were observed in all the prepared membranes, and in the spectra of the membranes with the insecticide cyantraniliprole, characteristic bands of the active molecule were observed, suggesting the interaction of the constituents of composition.

### 3.4 TGA-DSC

Figure 5 shows the thermograms of the prepared membranes containing different Lap and Alg ratios with and without the insecticide cyantraniliprole in their compositions. In the DTG curves of Figure 5 (A, B, and C), an initial event of an endothermic nature is observed, in the temperature range of 25 to 150 °C, where the respective mass losses occur, i.e., 18.1, 13.5, 13.7%, resulting from the dehydration of the membranes. Likewise, in Figure 5 (D, E, and F), with the presence of the insecticide cyantraniliprole, a mass decomposition event of an endothermic nature is observed, with mass losses of 8.8, 11.2, and 11.0%, respectively. Then, a second thermal decomposition event of an exothermic nature is observed in all samples, starting at 200 °C, resulting in respective mass losses of 31.9, 21.8, 19.6, 33.5, 30.7, and 17.2%. Analyzing the DTG curves, it is observed that the highest decomposition rate occurred around 240 °C, which can be attributed to the overlap of different simultaneous degradation processes of the clay, biopolymer and insecticide (when present in the composition of the material) and to the degradation of the mannuronic acid and guluronic acid chains that make up the structure of the Alg biopolymer. The third decomposition event, between 400 and 1000 °C, is predominantly endothermic and characterized by the decomposition of the aliphatic chain present in the Alg structure. At 1000 °C, the maximum heating temperature, the final residual mass was 27.5, 48.5, and 57. 8% for the samples T1, T2, and T3. Observing the thermograms with the presence of the insecticide cyantraniliprole (samples T4, T5, and T6), the mass loss events remained less pronounced, in descending lines from the temperature region of 400 °C.

**Figure 5:**
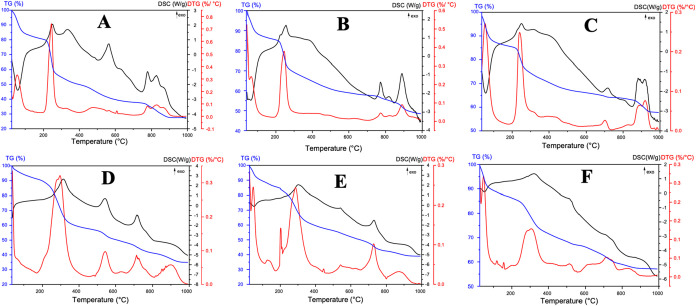
TGA-DSC curves membrane sample (A) T1 – Laponite RD 0.5% + sodium alginate 1.0%; (B) T2 – Laponite RD 1.0% and sodium alginate 1.0%; (C) T3 – Laponite RD 2.0% and sodium alginate 1.0%; (D) T4 – Laponite RD 0.5% and sodium alginate 1.0% + cyantraniliprole; (E) T5 – Laponite RD 1.0% and sodium alginate 1.0% + cyantraniliprole; (F) T6 – Laponite RD 2.0% and sodium alginate 1.0% + cyantraniliprole.

Through thermogravimetric and differential scanning calorimetric (TGA-DSC) analyses, it was possible to identify physical and chemical changes and energy variations in the polymeric membranes under study during the heating programs performed. Figure 5 shows that some thermal events were similar among the compositions evaluated, such as the first and second thermal events common to all prepared membranes. The first endothermic event suggested the elimination of water molecules adsorbed on the membranes, followed by the subsequent continuous release of water from the hydration spheres of exchangeable sodium cations in the Lap clay lamellae, and associated with the hydration of the alginate. The DTA curve of pure Alg showed initial water loss around 50 °C, while studies reveal that Lap/Alg nanocomposites show water evaporation at higher temperatures, close to 75 °C, a result like that of this study, around 60 °C.^12^ This suggests that the addition of Lap increases water retention in the nanocomposite. In addition, the clay acts as a physical barrier, delaying the manipulation of the polymer chain and shifting the resistance temperature values to higher levels, thus increasing the stability of the nanocomposite.

The DTG curves revealed peaks at temperatures above 600 °C, which may be related to the dihydroxylation of Lap clay and the formation of zinc and aluminum oxides.^24^ Studies indicate that above 600 °C, exchangeable cations migrate to the silicate layer, and the structure collapses, which may reveal exothermic peaks in this region.^24^ At the conclusion of the heating program, it was observed that the residual mass increased proportionally with the concentration of Lap. This finding suggests the inherent stability of the cationic clay, which is primarily attributed to the presence of silicon and magnesium oxides that resist rapid decomposition during heating.

### 3.5 SEM-EDS

SEM-EDS analyses examined the morphology, compatibility, and dispersion of Lap particles in the Alg biopolymer with and without the insecticide cyantraniliprole. The structural properties of clay particles are well-known to have a significant impact as reinforcements in polymer matrices, improving the physicochemical properties of the resulting composites. The incorporation of clays into polymers increases the elastic modulus, mechanical strength, stiffness, and swelling capacity and enhances biological activities.^25^ SEM images in Figure 6 show each composition, in which the white coloration between the platelets indicates the presence of clay dispersed within the Alg polymer matrix. In Figure 6A (see also Figure S1A for lower magnification), the composition is arranged in tactoids, i.e., in stacked layers, which may be related to the partial exfoliation process of Lap clay. As observed in the T1 diffractogram (Figure 3A green line), the material presents the characteristic peak of pure clay, referring to the diffraction of the atomic plane (001), in the region 2θ equal to 6.78°, which demonstrates the incomplete exfoliation of the clay. From the information obtained by the EDS technique (see Supporting Information), it is possible to state that the lamellae present a greater presence of Cl, Ca and F atoms, while the interlamellar spaces present significant amounts of Si, Na, Mg and aliphatic chains, highlighting the presence of 15% (w/w) of Na and 12% (w/w) of Si (Figure S2A,B). In addition, the high degree of alignment observed in Figures 6A and S1A suggests interactions between the edges of the Lap RD clay and the interconnected alginate structure, forming a network followed by a two-step deformation process. In the first step, water evaporation causes a change in the external volume of the entire microstructure without changing the orientation of the clay plates until a critical total solids concentration is reached, at which point the system develops a yield stress (Figure S2B). Once this yield stress is established, the Lap particles are immobilized, and subsequent drying aligns these plates.^26^ The whitish coloration observed in the SEM images may be related to the presence of clay nanoparticles in the membranes, which directly interferes with the mechanical properties and permeability of the material.^27^ This also indicates that Lap was dispersed in the polymer matrix of membranes.

**Figure 6:**
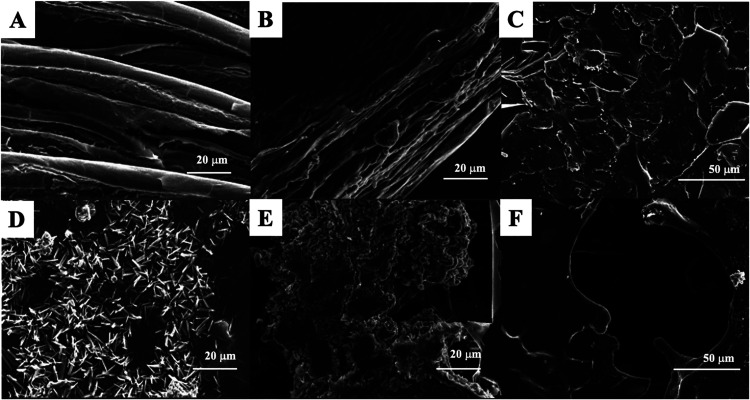
Representative SEM images of the membranes: (A) T1 – Laponite RD 0.5% + sodium alginate 1.0% at 3740× of magnification; (B) T2 – Laponite RD 1.0% and sodium alginate 1.0% at 3730× of magnification; (C) T3 – Laponite RD 2.0% and sodium alginate sodium 1.0% at 2250× of magnification; (D) T4 – Laponite RD 0.5% and sodium alginate 1% + cyantraniliprole at 3750× of magnification; (E) T5 – Laponite RD 1.0% and sodium alginate 1.0% + cyantraniliprole at 3750× of magnification; (F) T6 – Laponite RD 2.0% and sodium alginate 1.0% + cyantraniliprole at 3750× of magnification.

In Figure 6B (see also Figure S1B for lower magnification), a disorder of the composition can be observed, which suggests the exfoliation process of Lap, has already been observed in previous work.^28^ The diffractogram in Figure 3A (magenta line) validates the exfoliation process by the disappearance of the (001) peak in the 2θ region equal to 6.78°, characteristic of pure clay. Using the EDS technique, a uniform distribution of the elements in the material is observed, with the presence of Si and Mg standing out (Figure S3A,B). In Figure 6C (see also Figure S1C for lower magnification), the composition presents a lamellar texture and brittle character, again suggesting incomplete exfoliation of the clay, which is confirmed by the broad (001) peak at the beginning of the diffractogram in Figure 3A (red line). However, the EDS suggests a uniform distribution of the chemical elements over the membranes, with a greater presence, by mass, of Si and Mg (Figure S4A,B). This highlights the heterogeneous character of the membranes, partially exfoliated.

The images of compositions with the presence of the insecticide cyantraniliprole (Figure 6D–6F) (see also Figure S1D–F for lower magnification) revealed an abundantly porous structure. This can be justified by the interaction of the precursors (clay and biopolymer) with insecticide, which had already been described in pesticide preparations using nanocomposite hydrogels of polyacrylamide, montmorillonite, and alginate for controlled release of pesticides.^7^ With the porous structure obtained in the material containing the insecticide, combined with its own nature and method of application, these membranes do not possess the structural integrity necessary for conventional tensile tests. Moreover, the SEM images reveal that the incorporation of Lap (Figure 6D–6F) leads to the formation of rougher and more porous membrane surfaces compared to those without clay. This morphological pattern suggests that Lap particles are well dispersed throughout the polymeric matrix and do not hinder the cross-linking of alginate chains. With the addition of clay to the membranes, the spaces in the gels assume a flattened structure caused by the distribution of the lamellar structure in the pores, suggesting a good dispersion of the clay within the polymer matrix. As the amount of clay in the gel increases, the interaction and porosity within the gel network structure also increases.^7^ On the contrary, they appear to promote the formation of porous domains that may facilitate the diffusion of the active ingredient. The observed porosity and surface texture are favorable for controlled-release behavior, as they enable gradual water uptake and diffusion-mediated release of cyantraniliprole.

Using EDS, percentages of Cl were observed in all samples containing the active cyantraniliprole (Figures S5, S6, and S7). The crystallization of the insecticide can explain this during the drying process of compositions. In the case of T5, the binding of Cl atoms of the insecticide with Na atoms in the clay structure is observed, producing NaCl clusters within the structure (Figure S6). In T6 – Figure 6F (see also Figure S1F for lower magnification), the surface of the membrane creates interstices between the precursors,^29^ creating two groups according to the affinity of the atoms (Na, Cl, and S) and (Mg, Si and Ca) (Figure S7). In addition, the percentage amounts of chemical elements in the polymeric membranes, evidenced by the EDS technique, confirmed the chemical elements present in the chemical formulas of the precursors Lap (Na_0.7_ Si_8_Mg_5.5_Li_0.3_O_20_(OH)_4_) and Alg (C_6_H_7_NaO_6_) and cyantraniliprole.

### 3.6 Evaluation of Toxicity Bioassays on *L*. *coffeella*

For the study evaluating the corrected mortality of *L*. *coffeella* larvae 10 days after the application of polymeric membranes on *C*. *arabica* leaves, significant differences in larval mortality were observed: F­(6, 21) = 9.69, *p* < 0.001. The polymeric membranes that affected the mortality of *L*. *coffeella* larvae were T4, T6, T1, T5, and T7 (Figure 7A). Meanwhile, plants treated with T2 and T8 showed lower coffee leaf miner larva mortality. Treatment T3 was not included in the analysis due to the occurrence of phytotoxicity. Treatment T4 and T6 resulted in 93.4% and 92.9% mortality of larvae, respectively, while the control treatment T8 showed 6.6% mortality of larvae.

**Figure 7:**
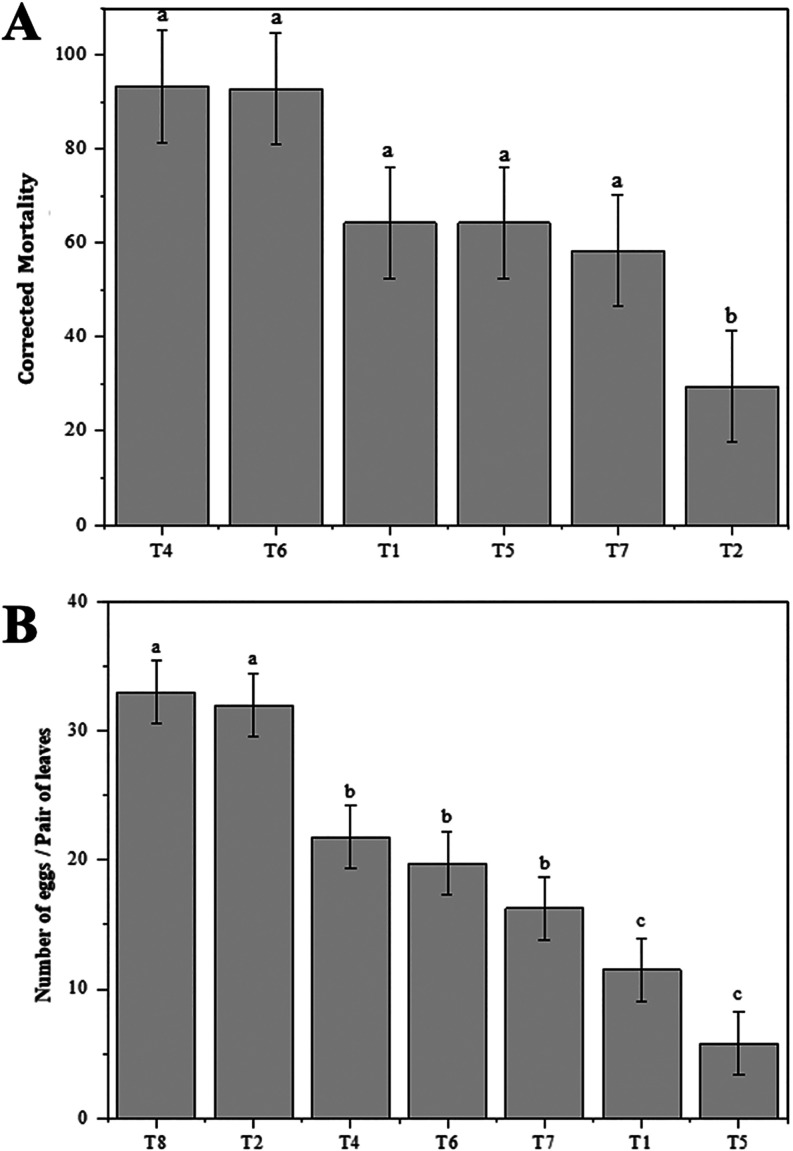
(A) Mean ± standard error of the corrected mortality of *Leucoptera coffeella* larvae per plant after the application of hybrid materials along with insecticides. Means followed by the same lowercase letter above the bars do not differ according to the Scott-Knott test (*p* < 0.05); (B) mean ± standard error of the number of *Leucoptera coffeella* eggs per plant after the application of hybrid materials along with insecticides. Means followed by different lowercase letters differ according to the Scott-Knott test (*p* < 0.05). T1 – Laponite RD 0.5% + sodium alginate 1.0%, T2 – Laponite RD 1.0% + sodium alginate 1.0%, T3 – Laponite RD 2.0% + sodium alginate 1.0%, T4 – Laponite RD 0.5% + sodium alginate 1.0% + cyantraniliprole, T5 – Laponite RD 1.0% + sodium alginate 1.0% + cyantraniliprole, T6 – Laponite RD 2.0% + sodium alginate 1.0% + cyantraniliprole, T7 – cyantraniliprole, T8 – Control. Treatment T3 was not included in the analysis due to the occurrence of phytotoxicity.

The membranes on *C*. *arabica* leaves showed a significant difference in the number of *L*. *coffeella* eggs per pair of leaves: F (6;21) = 16.93, *p* < 0.001. The polymeric membranes that most interfered with the oviposition of the coffee leaf miner were T5 and T1. Treatments T4, T6, and T7 also interfered with the oviposition behavior of the coffee leaf miner. Meanwhile, treatments T2 and the control T8 showed greater susceptibility to *L*. *coffeella* oviposition (Figure 7B).

The seedlings coated with treatments T4 and T6 presented larval mortality of 93.4 and 92.9%, respectively, and demonstrated promising results in repelling insect oviposition, with a reduction of 34.1 and 40.9% compared to the control. Additionally, treatments T5, T1, and T7 also presented reasonable larval mortality rates of 66.7, 66.7, and 61.1%, respectively, and demonstrated excellent results in repelling insect oviposition, with reductions of 82.6, 65.2, and 50.8% compared to the control. The lowest mortality and oviposition percentages among the selected membranes were observed in treatments T2 and control T8, which presented larval mortality rates of 34.2 and 6.6%, respectively, and the highest averages of eggs per pair of leaves. The results indicate that the presence of polymers on the upper part of the seedlings provides a physical and chemical barrier against *L*. *coffeella* adults, leading to insect mortality. Studies have demonstrated the association of Laponite with pyrethroids and neonicotinoids, which are contact and systemic insecticides.^30^ Both exposure routes may have contributed to larval mortality in *L*. *coffeella*, as the newly hatched larvae must feed on the leaf surface and penetrate the leaf tissue.^31^ Furthermore, it is likely that the membranes extend the control period for *L*. *coffeella*, since only the intercalation of Laponite with the analytical standard of chlorantraniliprole provided measurable mortality in an evaluation conducted 10 days after treatment application. This demonstrates the protective property of Laponite toward the insecticide molecule.^32^

The toxicity of cyantraniliprole is associated with its mode of action in insects. Cyantraniliprole, an anthranilic diamide insecticide, acts by targeting ryanodine receptors in insect muscle cells. This interaction leads to the depletion of intracellular calcium stores, ultimately causing paralysis and death.^33^ At sublethal doses, this insecticide can affect the physiological parameters of insects across various orders. It can reduce the levels of proteins, carbohydrates, and lipids, as observed in *Agrotis ipsilon* (Hufnagel, 1766) (Lepidoptera: Noctuidae).^34^ A reduction in body nutrient content can affect insect growth and the number of eggs per female.^35^ Studies on anthranilic diamide insecticides have shown significant negative effects on oviposition behavior and ovarial development in female lepidopteran pests.^36,37^ Several studies have demonstrated a reduction in the number of eggs per female treated with anthranilic diamide insecticides.^38–40^

Cyantraniliprole has been shown to prolong the insect life cycle and alter hormone levels.^41^ Although few studies have specifically examined the impact of cyantraniliprole on *L*. *coffeella*, research indicates that this pest has developed resistance to the diamide insecticide chlorantraniliprole; despite being a relatively recent insecticide class introduced in Brazil, continuous application in coffee-growing areas has selected for resistant populations.^42^ Moreover, rapid development of resistance to cyantraniliprole has been documented in the diamondback moth, *Plutella xylostella* L. (Lepidoptera: Plutellidae).^43^ Insecticide resistance in a pest population is primarily driven by factors such as increased application rates, repeated use of insecticides with the same mode of action, specific biological traits of the insect, and environmental conditions. The combination of these factors can accelerate the development of insecticide resistance.^44–48^

Thus, it was observed that coatings prepared based on Lap/Alg for coffee seedlings were effective alternatives to prevent insect oviposition, acting mainly as a physical barrier against the insect. The oviposition results suggest that the membranes can fill the microscopic pores of the insects’ antennae, making it difficult for them to identify the host. Furthermore, based on the results of larval mortality and oviposition, it can be stated that the membranes helped to interrupt the insect’s life cycle, i.e., altered the survival of *L*. *coffeella* at different stages of its life cycle, besides presenting higher rates than those presented by the commercial product used. There is scant literature documenting the association of Laponite with insect mortality. However, its use in combination with pyrethroid and neonicotinoid insecticides has been reported.^30^ Furthermore, there are reports of Laponite combined with oxamyl gel affecting larvae of the Colorado potato beetle, *Leptinotarsa decemlineata* (Say) (Coleoptera: Chrysomelidae).^49^ This evidence supports the premise that Laponite may have a synergistic effect with the insecticide chlorantraniliprole, or even extend the residual period of insecticides on the plant by providing greater stability to pesticide molecules.^30^

The addition of silicates to polymers improves the mechanical properties of the material and the adhesion of the surface membrane compared to pure polymers.^50,51^ This is due to the interactions between the silica present in the clay and the carboxyl groups of the polymer, which lead to cross-linking of the material and increased viscosity. However, high proportions of clay in the material increase opacity and promote the alignment of the layers, which reduces the gas barrier properties of the material. In addition, the emulsions and nanostructures present in the material allow a more effective coating of plant leaves than commercial pesticides, which suffer losses due to numerous processes such as volatilization, photolysis, degradation by hydrolysis, and environmental conditions. Compared to conventional formulations currently used to treat coffee leaf pests, such as emulsifiable concentrates directly sprayed onto leaves, the hybrid membranes developed in this study offer practical and environmental advantages reducing significantly application rates, protect against ultraviolet degradation, and allow the gradual release of substances, acting as a physical barrier against insect oviposition.

Moreover, these membranes adhere more effectively to the leaf surface and reduce active ingredient losses due to runoff or photodegradation. Their porous structure and heterogeneous morphology, as revealed by SEM, promote a sustained and gradual release of cyantraniliprole, thereby enhancing insecticidal efficacy over time. Although *in vitro* release tests under different solvent conditions were not performed in this study, the increased porosity observed with Lap incorporation supports the hypothesis that the release is governed by diffusion through the polymer matrix. Thus, the proposed system not only contributes to a longer-lasting field effect but also reduces the frequency of application and potential environmental contamination, aligning with sustainable pest control practices.

## 4 Conclusions

Hybrid membranes have emerged as a promising alternative for the sustainable management of the coffee leaf miner in coffee plantations. XRD and FTIR-ATR analyses confirmed the presence of organic and inorganic precursors, as well as the synergistic interaction between the membranes and the insecticide cyantraniliprole, through the decrease in intensity and disappearance of peaks and bands, respectively, corroborating the inference that the obtained hybrid system facilitates the gradual release mechanism of the insecticide. TGA-DSC analysis verified the thermal stability of the membranes, revealing a main decomposition event between 150 and 400 °C, attributed to molecular dehydration. SEM-EDS analysis showed a mixture of organic and inorganic components, increased material porosity with the incorporation of the insecticide, and the presence of O, Na, Mg, and Si in the polymeric membranes, forming a diffusion barrier capable of modulating the transport of the active ingredient to the leaf environment. In bioassays, the use of organic–inorganic hybrid materials as protective membranes on coffee leaves proved more effective in controlling *L*. *coffeella* than the standard commercial product. Greenhouse experiments demonstrated that membranes applied to coffee leaves significantly increased larval mortality and repelled *L*. *coffeella*, with some treatments reaching up to 93.4% larval mortality. Furthermore, some membranes without insecticides in their composition also showed significant efficacy, suggesting that the combination of these precursors can establish an effective physical barrier for the insect, reducing application volumes and the excessive use of conventional insecticides.

The use of new technologies, such as Laponite, can optimize insecticide formulation. Through controlled-release mechanisms, it may be possible to reduce both the number of applications and the effective doses required for pest control. This approach minimizes the adverse effects of these compounds on the environment and nontarget organisms, thus promoting greater economic, social, and environmental sustainability.

Among the limitations observed in the study are the absence of kinetic curves to evaluate the insecticide release rate and the mechanical characterization of the hybrid membranes. Future studies should focus on characterizing the release kinetics under simulated environmental conditions and evaluating the long-term performance of these membranes in field tests to confirm the controlled release of the active ingredient cyantraniliprole evidenced by SEM and XRD analyses. Regarding the mechanical characterization of hybrid membranes, the development of formulations that support standardized mechanical tests will provide a more comprehensive understanding of the stability and performance of these materials.

Taken together, these findings highlight the potential of hybrid organic–inorganic membranes as an innovative and sustainable strategy for the management of Leucoptera coffeella in coffee cultivation. By integrating structural, thermal, and morphological evidence with promising biological responses, the study demonstrates that these materials can not only enhance the efficiency of cyantraniliprole through controlled release but also act as effective physical barriers capable of reducing the reliance on conventional insecticide applications. Although additional investigations—particularly regarding release kinetics, mechanical performance, and long-term field validation—are still required, the results presented here establish a solid foundation for the advancement of hybrid membrane technologies and their future application in precision pest management within agroecosystems.

## Supplementary Material



## Notes

Additional results regarding the description of the proportions of the polymeric membranes used in the coating test; representative SEM images of the membranes at lower magnifications; EDS analysis of membranes T1–T6 (PDF)
