# Supplementary material for: Enhanced Control of Leucoptera coffeella on Coffee Leaves Using Cyantraniliprole-Hybrid Polymeric Membranes
Source: ACS Omega. 2026 Jan 23;11(4):6671–82. doi: 10.1021/acsomega.5c11821 (PMC12878367; doi:10.1021/acsomega.5c11821)
Supplement: Supplementary file 1 [file ao5c11821_si_001.pdf]

Enhanced Control of *Leucoptera coffeella* on Coffee Leaves using Cyantraniliprole-Hybrid Polymeric Membranes

Caroline Nunes dos Reis[a], Lorena Alves de Melo Bessa[a], Maria Gabrielle Silva[a], Thaissa Moreira Santos[a], Keyller Bastos Borges[b], Eduardo Alves[c], Júlio César José da Silva[d], Gustavo Franco de Castro[e], Carlos Gustavo da Cruz [a], Flávio Lemes Fernandes[a], and Jairo Tronto[a]

[a] Instituto de Ciências Exatas e Tecnológicas, Universidade Federal de Viçosa, Campus Rio Paranaíba, 38810-000, Rio Paranaíba, Minas Gerais, Brazil.

[b] Departamento de Ciências Naturais, Universidade Federal de São João del-Rei (UFSJ), Campus Dom Bosco, Praça Dom Helvécio 74, Fábricas, 36301-160, São João del-Rei, Minas Gerais, Brazil

[c] Universidade Federal de Lavras, Campus Lavras, 37200-000, Lavras, Minas Gerais, Brazil

[d] Departamento de Química Instituto de Ciências Exatas (ICE) Universidade Federal de Juiz de Fora (UFJF), Campus Universitário, s/n, Bairro Martelos, Juiz de Fora -Minas Gerai, Brazil

[e] Universidade Federal de Viçosa, Campus Viçosa, 36570-900, Viçosa, Minas Gerais, Brazil.

Correspondence:

Prof. Jairo Tronto, Ph.D. Instituto de Ciências Exatas e Tecnológicas, Universidade Federal de Viçosa, Campus Rio Paranaíba, CEP 38810-000, Rio Paranaíba, MG, Brazil.

e-mail: jairotronto@ufv.br

Tables

Table S1. Description of the proportions of the polymeric membranes used in the coating test.

Lap 0.5% + Alg 0.5%	Lap 0.5% + Alg 1.0%	Lap 0.5% + Alg 2.0%
Lap 1.0% + Alg 0.5%	Lap 1.0% + Alg 1.0%	Lap 1.0% + Alg 2.0%
Lap 2.0% + Alg 0.5%	Lap 2.0% + Alg 1.0%	Lap 2.0% + Alg 2.0%
Lap 3.0% + Alg 0.5%	Lap 3.0%+ Alg 1.0%	Lap 3.0%+ Alg 2.0%

Figures

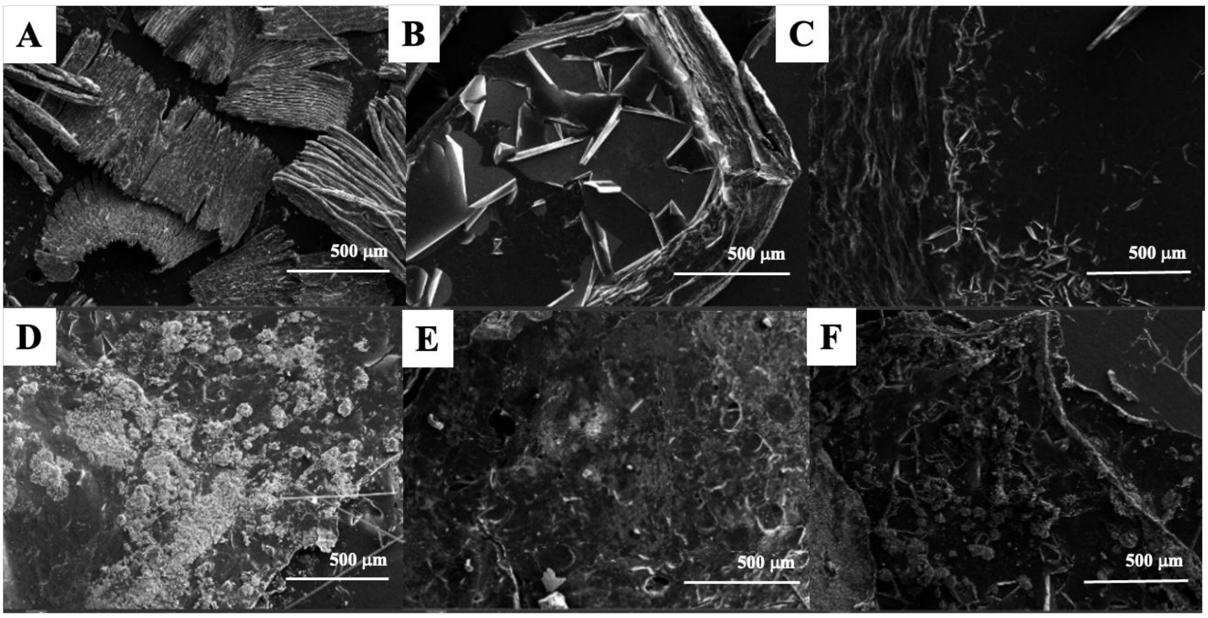

Figure S1. Representative SEM images of the membranes (A) T1 - Laponite® RD 0.5% + sodium alginate 1.0% at 225 × of magnification; (B) T2 - Laponite® RD 1.0% and sodium alginate 1.0% at 230 × of magnification; (C) T3 - Laponite® RD 2.0% and sodium alginate 1.0% at 225 × of magnification; (D) T4 - Laponite® 0.5 % and sodium alginate 1.0% + cyantraniliprole at 224 × of magnification; (E) T5 - Laponite® RD 1.0% and sodium alginate 1.0% + cyantraniliprole at 246 × of magnification; (F) T6 - Laponite® RD 2.0% and sodium alginate 1.0% + cyantraniliprole at 217 × of magnification.

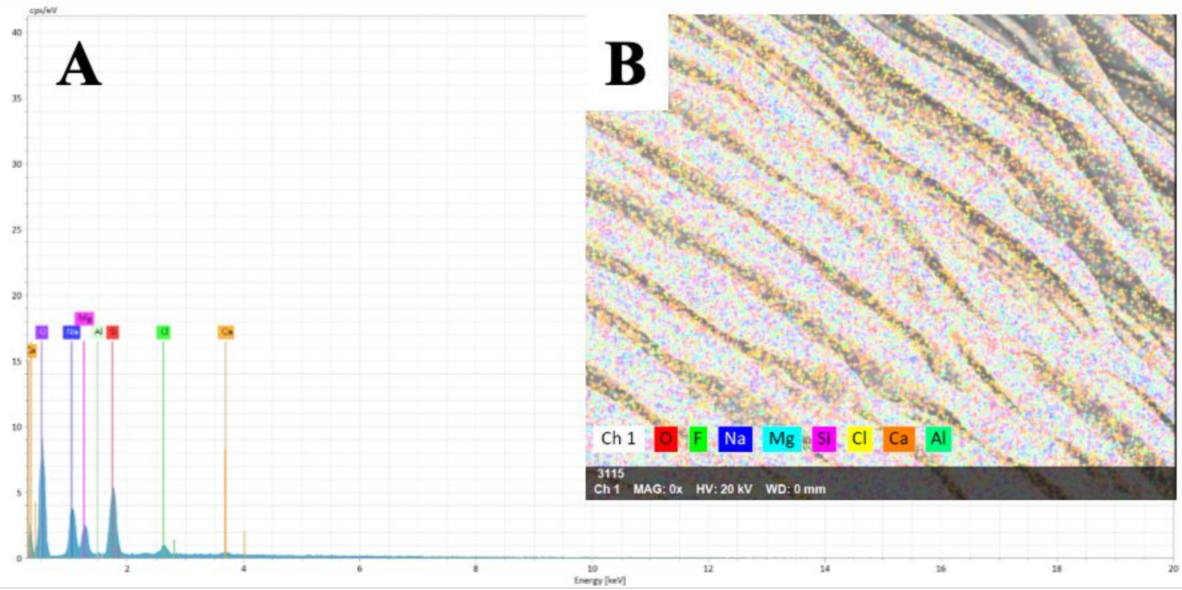

Figure S2. (A) EDS qualitative percentage of elements in T1 sample; (B) EDS images of the membranes T1 - Laponita® RD 0.5% + sodium alginate 1.0%.

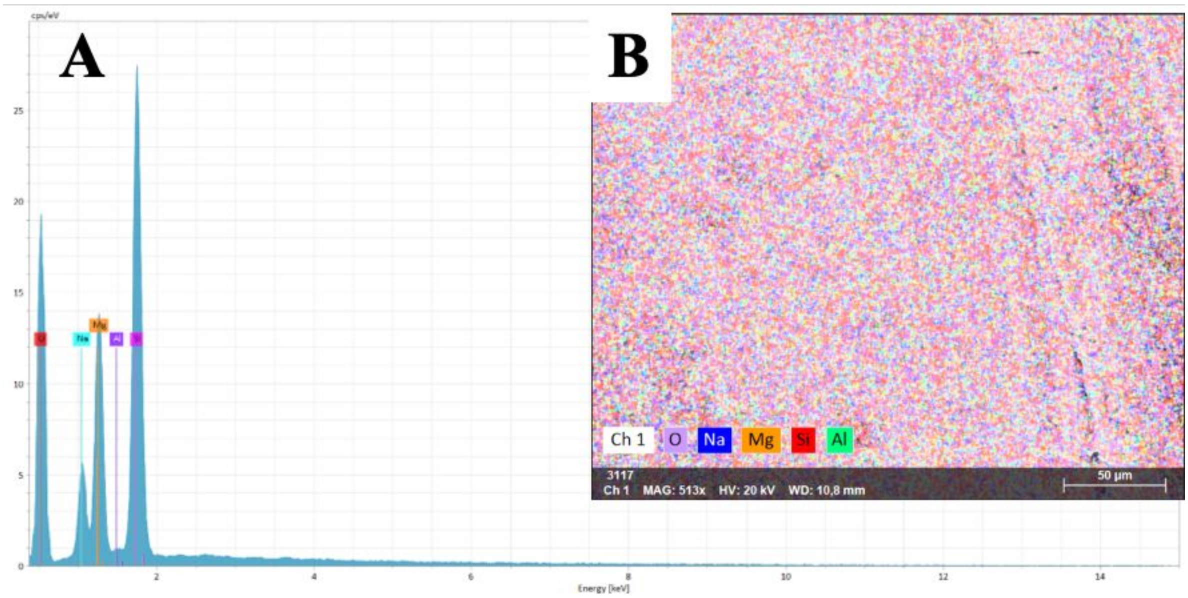

Figure S3. (A) EDS qualitative percentage of elements in T2 sample; (B) EDS images of the membranes T2 - Laponita® RD 1.0% + sodium alginate 1.0%.

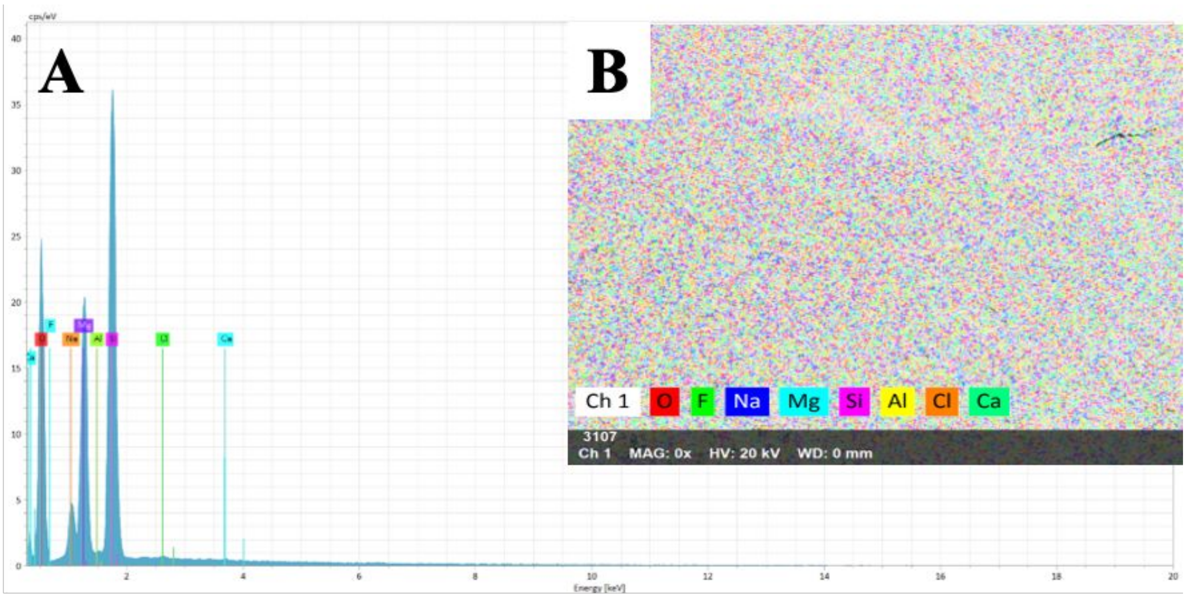

Figure S4. (A) EDS Qualitative percentage of elements in sample T3; (B) EDS images of the membranes T3 - Laponita® RD 2.0% + sodium alginate 1.0%.

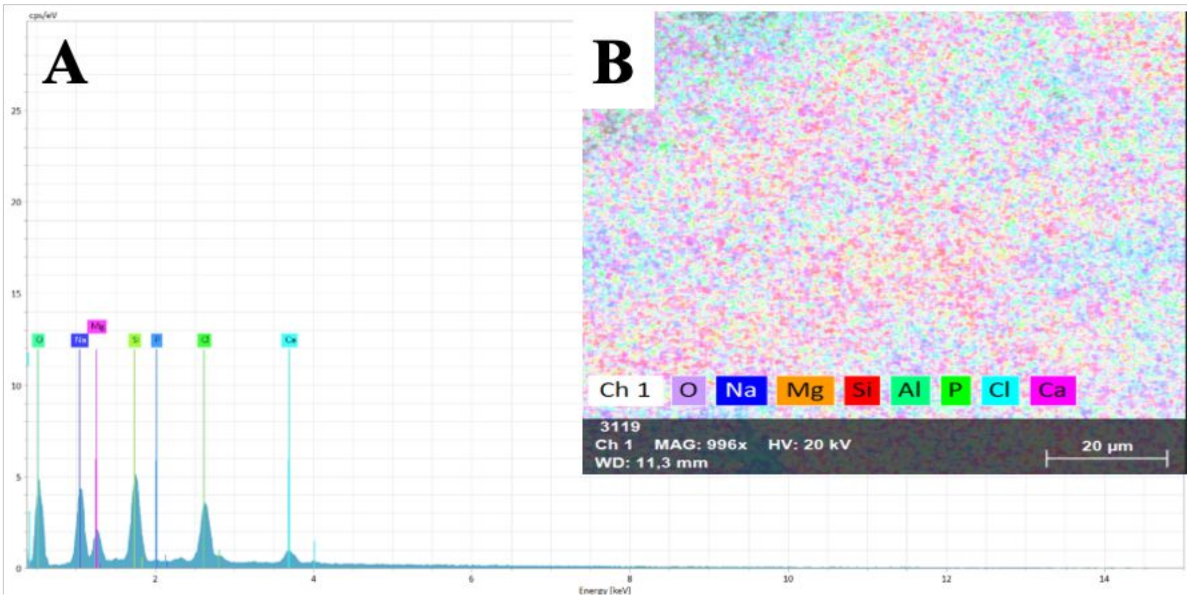

Figure S5. (A) EDS qualitative percentage of elements in T4 sample; (B) EDS images of the membranes T4 - Laponita® RD 0.5% + sodium alginate 1.0% + cyantraniliprole.

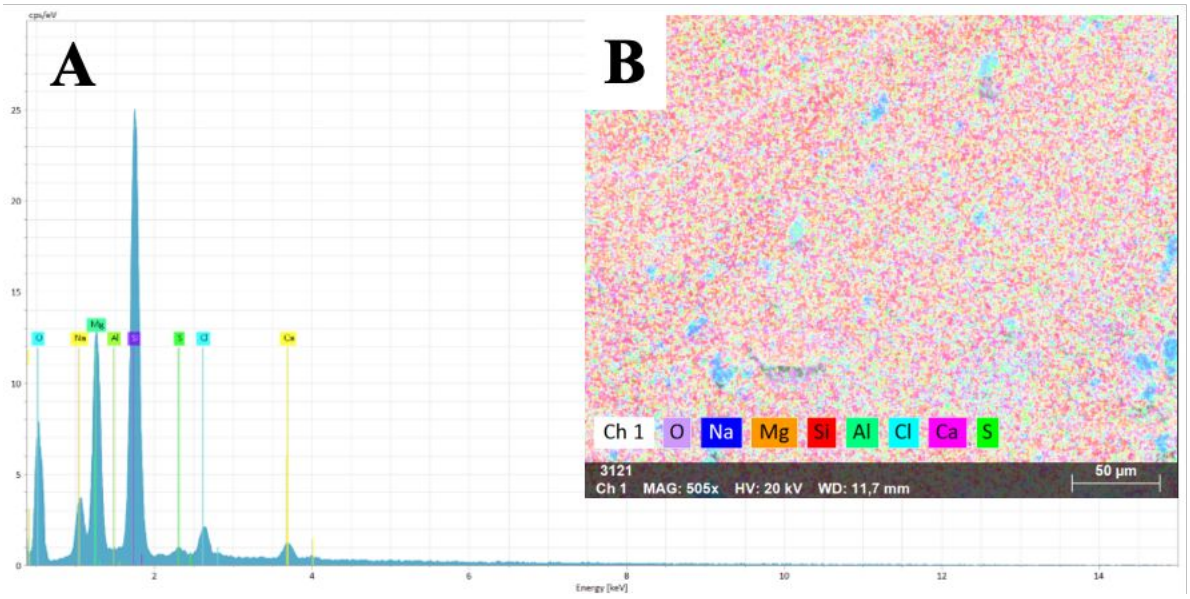

Figure S6. (A) EDS qualitative percentage of elements in T5 sample; (B) EDS images of the membranes T5 - Laponita® RD 1.0% + sodium alginate 1.0%+ cyantraniliprole.

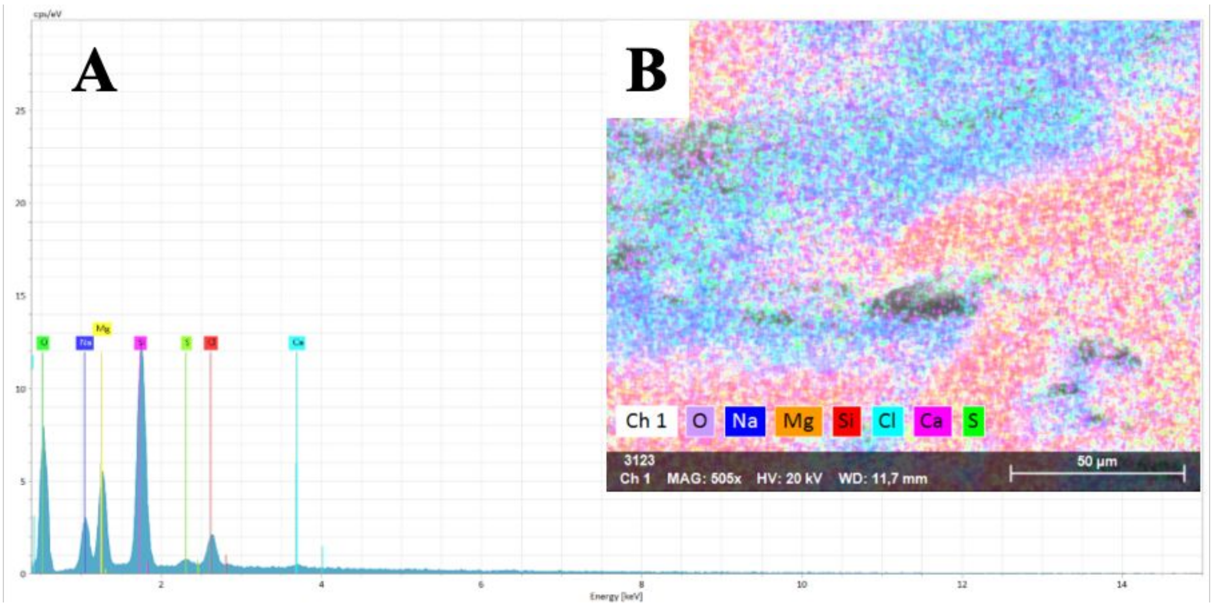

Figure S7. (A) EDS Qualitative percentage of elements in sample T6; (B) EDS images of the membranes T6 - Laponita® RD 2.0% + sodium alginate 1.0% + cyantraniliprole.
